# Clinical aspects and recent advances in fungal diseases impacting human health

**DOI:** 10.1093/jac/dkaf004

**Published:** 2025-03-14

**Authors:** Livio Pagano, Omar Marín Fernández

**Affiliations:** Dipartimento di Diagnostica per Immagini, Radioterapia Oncologica ed Ematologia, Università Cattolica del Sacro Cuore, Rome, Italy; Fondazione Policlinico Universitario Agostino Gemelli – IRCCS, Rome, Italy; F2G Ltd, Eccles, Manchester, UK

## Abstract

Fungal diseases are of growing clinical concern in human medicine as the result of changes in the epidemiology, diversity in clinical presentation, emergence of new pathogens, difficulties in diagnosis and increasing resistance to antifungals of current available classes. There is a need for high disease awareness among the public and healthcare physicians, improvement in diagnostic methods and the development of drugs from new therapeutic classes with an improved resistance profile. In this article, we will explore some key aspects of fungal diseases in humans and provide a general overview of this important topic.

## Introduction

Fungi cause diverse infections in humans that together affect more than a billion people globally, with severities ranging from allergies to life-threatening invasive fungal infections (IFIs).^1^ The burden of fungal infections, particularly IFIs, is a growing public health problem, which has been exacerbated by the emergence of new fungal pathogens and resistant strains, and the resurgence of previously uncommon diseases.^1,2^ While it is difficult to measure true prevalence partly due to challenges with diagnostics, a recent review estimated an annual incidence of 6.5 million IFIs and approximately 2.5 million attributable deaths, highlighting the global burden of these infections.^3^

Understanding the nature of fungal infections, their causes, symptoms, and available treatments is crucial for effective prevention and treatment.^4^

## Specific challenges of fungal infection in humans

**Figure 1.**
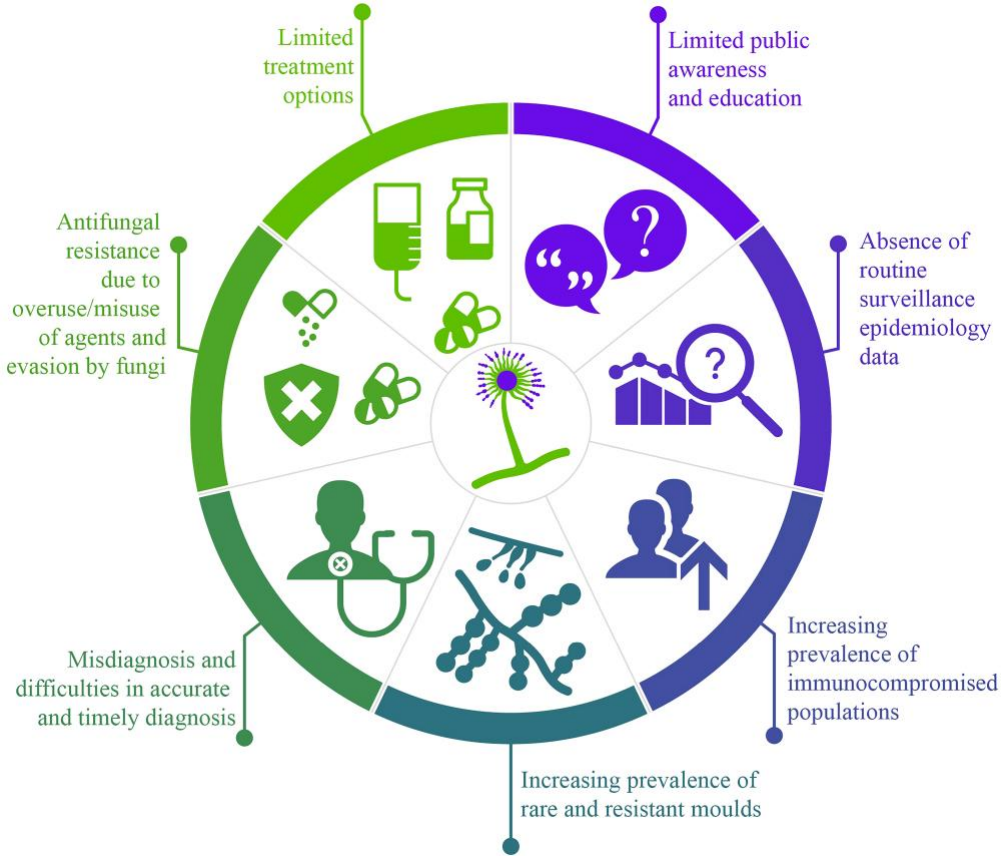
Challenges in the management of IFIs.

Fungal infections pose significant challenges (Figure 1) due to the increasing incidence and diverse nature, including highly resistant strains, which can complicate prevention, diagnosis and treatment.^2^ Effective treatment options are limited, and this can be further complicated by resistance and patient characteristics such as immunosuppression.^5–7^ Improving awareness of these challenges is important to prevent any further hindrance to effective diagnosis and treatment of patients with IFI.^4^

Some of the key challenges in dealing with fungal infections are described below.

### Limited public awareness and education

Many fungal infections are relatively uncommon compared with other infectious diseases, leading to limited awareness among healthcare professionals and the public.^4^ This lack of awareness can result in delayed diagnosis, inadequate treatment and a higher risk of disease transmission.^4^ Raising awareness, promoting education and improving healthcare providers' knowledge about fungal infections are crucial for early detection and effective management.^4^

### Misdiagnosis and difficulties in diagnosis and susceptibility testing

Maximizing the survival to fungal infections requires readily available guidance to allow rapid diagnostic and therapeutic intervention.^8^ Fungal infections can often be mistaken for other types of infections, such as bacterial or viral infections, or the type of IFI may be misdiagnosed; this can lead to inappropriate treatment (e.g. empiric treatment with an ineffective agent), which may contribute to the emergence of antifungal resistance, prolong illness or complications and increase mortality rates.^9–12^

Conventional culture methods remain the gold standard for diagnosing fungal infections; however, these tests have relatively low sensitivity, can be slow and labor intensive and rely on mycologists with expertise in phenotypic identification.^12^ Even so, fungal species (particularly rare fungi) can be difficult to differentiate, and microscopic identification can be subjective.^13^ Antifungal susceptibility testing methods have greatly improved over recent years, yet continue to be associated with high variation between laboratories and methods used; therefore, standardization, validation and extensive training are essential for accurate interpretation and translation into clinical decision making.^13^ Many fungal species, particularly invasive molds, are lacking clinical breakpoints and instead rely on epidemiologic cut-off values, further complicating the accurate interpretation of results.^14^ Furthermore, dedicated clinical mycologists and mycology laboratories are not available at every centre and in some countries are not available at all due to limitations in infrastructure and funding.^12^ Indeed, some fungal species are unable to be cultured, thus evading conventional detection.^12^

Advances in fungal diagnostics have yielded several faster, more sensitive diagnostic approaches that are either in use or under investigation, including novel PCR assays, microfluidic chip technology, next-generation sequencing, biosensor-based technologies and artificial intelligence–based models.^12,15^ All of these technologies aim to improve the speed and accuracy of fungal diagnostics to reduce delays to treatment and improve patient outcomes.^12,15^ Promisingly, molecular testing has now been incorporated into the second revision of the European Organisation for Research and Treatment of Cancer (EORTC)/Mycoses Study Group Education and Research Consortium (MSGERC) definitions for invasive fungal disease; this is owing to improvements in commercial PCR assays, such as a greater ability to directly detect fungi in clinical specimens and findings of clinical validation studies.^12,16^ However, extensive validation data on larger patient cohorts in well-designed multicentre studies are required for newer methods to ensure they can be incorporated into diagnostic settings.^12^

### Rise in IFI caused by rare and resistant molds

IFIs are a group of infections that can affect different organs or systems, including the lungs, bloodstream, CNS and skin.^17^ These infections are especially problematic in immunocompromised patients, as well as in critically ill patients who are not immunocompromised, where they are associated with high mortality rates without adequate and timely therapy.^18,19^

The true global incidence and epidemiology of IFIs is difficult to determine due to a lack of studies and surveillance data.^20^ Monitoring the epidemiology of these infections is complicated by the high amount of variation across different geographical regions, likely due to differences in clinical practices, availability of diagnostic techniques and differences in patient populations.^21^ However, the burden of IFIs is only likely to worsen over the coming years given the growing population of susceptible patients such as those undergoing invasive procedures, enduring long ICU stays and suffering from immunosuppressive conditions (e.g. solid organ and stem cell transplantation).^20,21^ This burden is further compounded by the rise in antifungal resistance, with azole-resistant *Aspergillus* and *Candida* spp. increasingly reported worldwide.^22^

The most common pathogens associated with IFIs are *Candida*, *Aspergillus*, *Cryptococcus* and *Pneumocystis* spp., and these are the causal pathogens in over 90% of reported deaths due to IFI.^23^ Mycoses caused by rare molds such as *Fusarium*, *Lomentospora*, *Rasamsonia*, *Scopulariopsis*, *Penicillium*, *Talaromyces* spp. other than *Talaromyces marneffei*, *Paecilomyces*, *Purpureocillium* and *Schizophyllum* spp. are also increasing,^24^ but mold fungi from the genera *Aspergillus* and *Scedosporium* represent particular threats especially to immunosuppressed individuals.^25^

Invasive aspergillosis (IA) is a rapidly progressive, often fatal infection, with reports of genetically distinct *Aspergillus* spp. with intrinsic resistance mechanisms.^26,27^ The triazole antifungals are the standard of care for these fungi infections; however, infections with mutational resistance to triazoles often leave patients with limited treatment options.^28^

Mucormycosis is the second most prevalent mold infection after IA and can occur in patients with hematological malignancies and transplant recipients.^29^ Incidence has risen globally over recent decades, associated with the COVID-19 pandemic, as well as the increasing immunocompromised population, higher incidence of diabetes and improved detection methods.^30^ Effective treatments for mucormycosis are lacking due to its high propensity for resistance to many antifungal agents, often resulting in treatment delays and poor prognoses for patients.^31^

Invasive scedosporiosis (IS) is less frequent than IA; however, there are increasing numbers of cases being reported.^32^ Nevertheless, *Scedosporium* spp. infections have high therapeutic failure rates and often result in relapse.^33,34^*Lomentospora prolificans* is a species that is pan-antifungal resistant and represents a particular threat to both immunocompetent and immunocompromised patients, in whom mortality rates reach up to 87%.^35,36^

Invasive fusariosis (IF) is an uncommon but severe infection, predominately affecting immunocompromised patients with prolonged neutropenia, underlying hematological malignancy, and patients undergoing hematopoietic stem cell transplantation.^37–39^ Disseminated fusariosis is highly challenging to treat and associated with high mortality rates of 66%–75%.^39^*Fusarium* spp. demonstrate high rates of intrinsic resistance to most antifungal agents, and resistance among individual species is highly variable, making early identification of pathogenic strains and susceptibility profiling essential for treatment.^40^

### Environmental sources of fungal infection

Fungal spores are ubiquitous in the environment and can be found in soil, plants, water and even in indoor spaces.^41^ This makes it challenging to completely avoid exposure to fungal pathogens. Certain occupations, such as farming or construction work, can increase the risk of fungal infections due to frequent contact with contaminated environments.^42^ For some endemic mycosis (coccidioidomycosis), there has been a steady increase in reported cases year over year during the last decade.^43^ This increase may be partly due to increased populations, soil disturbance (construction) and climate change.^2,41,43^

### Increasing prevalence of immunocompromised and other susceptible patient populations

In recent years, the proportion of immunocompromised patients has risen to roughly a third of all ICU admissions.^44^ The greater use of solid organ and hematopoietic stem cell transplantation, more aggressive and prolonged cancer treatments and the introduction of steroid-sparing agents to treat auto-immune and auto-inflammatory diseases are all contributing factors to this increase in immunocompromised patients.^44^

Individuals with weakened immune systems, such as those with HIV/AIDS, hematologic or solid cancers, organ transplants or auto-immune diseases, are more susceptible to fungal infections and often have more severe disease courses.^44,45^ Treating fungal infections in immunocompromised patients poses additional challenges due to factors such as drug-to-drug interactions (DDIs), drug toxicity and compromised immune responses.^6,7^

Patients not classically defined as immunocompromised, such as those in an ICU setting, critically ill patients with influenza or COVID-19 and patients receiving therapies for inflammation (e.g. TNF-α inhibitors), are increasingly identified as being at risk of developing IA.^6,46,47^

Patients receiving tyrosine kinase inhibitors (i.e. FMS-like tyrosine kinase 3 [FLT-3] inhibitors) or B-cell lymphoma 2 (BCL-2) inhibitors for acute leukemia are of particular concern: these potent chemotherapy agents can exhibit substantial DDIs with azoles, typically used either to prevent or treat IA.^48^ Thus, clinicians face a difficult choice between maintaining both effective chemotherapy and maximal antifungal dosing.

### Nature of fungal infections

Fungal infections can be chronic in nature and persist for prolonged periods, such as chronic pulmonary aspergillosis, which generally affects immunocompetent patients with pre-existing pulmonary comorbidities.^49^ Infections may also be characterized as frequently recurrent, such as allergic bronchopulmonary aspergillosis, which is an *Aspergillus fumigatus* hypersensitivity disorder in mostly patients with asthma or cystic fibrosis.^50^

Recurrence can be attributed to the ability of fungi to form protective structures called biofilms, which make them resistant to treatment and allow immune evasion.^51^ Furthermore, fungi have developed a variety of other virulence factors that allow species to adapt to the host environment and alter fitness in a multifactorial manner; these include metabolite production, cell wall maintenance, managing immune system interactions and tolerance to stress.^52,53^

### Antifungal resistance

Many fungal species have developed resistance to all four classes of antifungal drugs, i.e. polyenes, triazoles, echinocandins and the pyrimidine analogue 5-flucytosine, and a few fungal strains are intrinsically resistant to these antifungal agents.^54,55^

Antifungal resistance can arise due to various factors; common factors in mold infections include overuse or misuse of antifungal agents, inadequate dosing and improper patient adherence to treatment.^10^ The use of azoles in agriculture to control fungal plant infections has significantly contributed to improving crop yield and quality but has led to the development of fungicide resistance and the transmission of *A. fumigatus* strains with resistance to the azole class.^56^

Antifungal resistance complicates the effectiveness of treatment, leading to prolonged illness, increased healthcare costs and higher mortality rates, as it reduces the effectiveness of available treatment options and may require the use of more toxic or expensive antifungal drugs.^57,58^

### Limited antifungal treatment options

Often considered secondary to bacterial or viral infections, fungal infections have relatively limited treatment options.^5^ This scarcity of antifungal agents is further exacerbated by the emergence of new pathogenic fungal species and the limited development of novel antifungal drugs.^5,59^

There are three major classes of antifungals in the market: triazoles, which target the cell membrane by inhibiting ergosterol synthesis (e.g. voriconazole, posaconazole, isavuconazole); echinocandins, which inhibit the synthesis of (1,3)-β-D-glucan (e.g. caspofungin, micafungin, anidulafungin); and polyenes, which bind to ergosterol and disrupt membrane integrity, such as deoxycholate amphotericin B (AmB) or lipid formulation of AmB.^54^

Current early antifungal treatment strategies include prophylaxis and pre-emptive therapy, which often result in long-term exposure to antifungal agents and drive resistance mechanisms due to selective pressure.^60^ The increasing incidence of azole-resistant *Aspergillus* and *Candida* spp. strains is of concern as triazoles have been the gold standard treatment in life-threatening IA and candidiasis for two decades.^61–63^ The IS treatment options are even more limited with inherent resistance to polyenes and no current mediator with reliable activity against *L. prolificans*.^32,36^ Antifungals are further limited by limited dosage forms, DDIs and significant adverse reactions.^61^

As a result of treatment limitations, some fungal infections require either combination therapy or have no effective treatment, leaving patients at a higher risk of complications or death.^24,32,36^ International guidelines provide recommendations on combination therapies for certain fungal infections: anidulafungin plus voriconazole is a recommended option for azole-resistant IA by the European Society of Clinical Microbiology and Infectious Diseases, European Confederation of Medical Mycology (ECMM) and European Respiratory Society;^64^ and ECMM, the International Society for Human and Animal Mycology and the American Society for Microbiology recommend liposomal AmB (L-AmB) plus flucytosine for cryptococcosis,^65^ voriconazole- or L-AmB-based combination therapy for IF and voriconazole-based combination therapy for infection caused by *L. prolificans*.^24^

### New antifungals and clinical trials

The development of new antifungal agents is complicated by difficulties faced in conducting large clinical trials.^66^ IFIs are rare in comparison with other infectious diseases; therefore, patient numbers are often low and recruitment is challenging.^66,67^ The invasive nature of testing is not suitable for all patients, and populations at risk are highly variable, so stratifying patients is particularly complicated.^66,67^

Despite these challenges, several new antifungal agents have emerged in recent years, three of which have now been approved by the Food and Drug Administration Agency, including ibrexafungerp in June 2021, oteseconazole in April 2022 and most recently, rezafungin in March 2023.^68^ Further novel antifungal agents are under investigation in clinical trials, including olorofim and fosmanogepix.

Olorofim is a first-in-class orotomide antifungal, which selectively inhibits fungal dihydroorotate dehydrogenase, a key enzyme in fungal pyrimidine biosynthesis.^69,70^ Olorofim demonstrates potent activity against most clinically relevant *Aspergillus* spp., including azole-resistant strains, and activity against difficult-to-treat resistant molds (e.g. *Lomentospora* spp.) and dimorphic fungi (e.g. Coccidioides).^69–71^ Currently, olorofim is under Phase 3 evaluation for the treatment of IFI caused by *Aspergillus* spp. in patients with limited treatment options.^72^

Fosmanogepix is a prodrug of manogepix that inhibits the fungal acyltransferase enzyme (Gwt1) and disrupts the formation of the fungal cell wall and membrane.^69^ With its novel mechanism of action, fosmanogepix demonstrates broad-spectrum antifungal activity against most *Candida*, *Cryptococcus* and *Aspergillus* spp., as well as other rarer molds such as *Fusarium* and *Scedosporium* spp.^69,73^ Fosmanogepix is planned to undergo Phase 3 clinical evaluation for the treatment of candidemia and/or invasive candidiasis.^74^

These new antifungal agents provide potential alternative treatment options for IFIs, which is particularly important considering increasing rates of antifungal resistance, including cross resistance between antifungals of the same class. While more clinical data on the safety and efficacy of these agents emerge, it will also be important to consider the implementation of stewardship programmes and increased surveillance, including utilization in agriculture, in order to slow resistance development and ensure the longevity of new treatments.

## Summary

Addressing these challenges requires a multi-faceted approach that includes an increased awareness, improved diagnostic techniques, better surveillance, responsible use of antifungal medications and targeted interventions for high-risk populations. By understanding and addressing these challenges, we can enhance the management and prevention of fungal infections in humans, ultimately improving the overall health and well-being of individuals affected by these infections.

There is an unmet clinical need for an antifungal agent with a wide range of activity and novel mechanism of action against molds, including molds that have developed resistance to current antifungals and those that are intrinsically resistant, for which the optimum therapeutic approach is currently not clear.

Increasing resistance mechanisms exacerbated by increasing numbers of immunocompromised patients and limited alternative treatment options, indicate an urgent medical need for the development of newer antifungals with high efficacy against a comprehensive range of fungal species.
